# Interstate Migration of Tuberculous Persons

**Published:** 1915-10

**Authors:** 


**Interstate Migration of Tuberculous Persons.** Ernest A. Sweet, Reprint No. 269, Pub. Health Reports, mentions Texas, New Mexico, Arizona, Colorado and California as the principal states into which the tuberculous have immigrated, the first four having practically the same climatic advantages, due to high elevation, average of sunshine and low precipitation. Winds and dust, but rarely smoke, are practically the only objectionable features. Contrasted with an average tuberculous death rate of 149.5 : 100,000 population, Albuquerque has a like mortality of about 1400; El Paso of about 800; Colorado Springs of 600; San Antonio of 450; Denver of 350; San Diego of 300; Los Angeles of 300. On the whole, the tuberculous death rate has diminished, as states and cities have developed industrially, the tuberculous population being, so to speak, diluted with a normal population. Of 1419 tuberculous deaths at Albuquerque, 7.8% were of natives. At El Paso, 12.1% of 2,791; At San Antonio, 33.3% of 4,180. Obviously, these statistics are liable to fallacies in both directions: a large proportion of the population of western states were born in the east, while heredity—or rather familial infection—and infection from immigrants, undoubtedly causes much of the tuberculosis in the native born or permanent residents. Albuquerque has had about 2,000 tuberculous immigrants from other states per year, for ten years; El Paso about 3,000, San Antonio about 3,000.

One of the saddest features of the problem is the unnecessary suffering due to the migration of hopeless cases. Five deaths on trains bound for Albuquerque occurred in 1912, and the average for the ten years, 1904-13, 15.6% occurred within 30 days, 30.9% more within 6 months. For El Paso, the figures are: 2,791, 11.5% and 19%. For San Antonio, of 4,180, 32.7% died within 6 months of arrival.

The burden imposed upon western municipalities by the indigent tuberculous immigrants is considerable. As previously mentioned, place of birth is scarcely a criterion, but it appears that something like 80% of the indigent tuberculous population are supported by these municipalities unjustly.

The report enters into methods of car disinfection and discusses various other phases of the general problem.

